# 

**Published:** 1977

**Authors:** 


Bristol Medico-Chirurgical Journal Vol. 92. No. 341/342

### Contents

Cancer and Mental Retardation ... 3

M. P. Jancar and J. Jancar

Mycoplasma Pneumonia in Adults ... 9

Roger White

The Burden Heritage ... ... 12

Dr. D. A. Primrose

Printed by F. Bailey & Son Ltd., Dursley, Glos.

